# Severe Rosuvastatin-Associated Myopathy With Persistent HyperCKemia and Transaminitis: Early Outpatient Recognition and Diagnostic Pitfalls

**DOI:** 10.7759/cureus.113256

**Published:** 2026-07-23

**Authors:** Hussain Ahmad Bhatti

**Affiliations:** 1 Medicine, Clinica Portada, Antofagasta, CHL

**Keywords:** case report, creatine kinase, drug-induced myopathy, hyperckemia, inflammatory myopathy, proximal muscle weakness, rosuvastatin, statin-associated myopathy, statin toxicity, transaminitis

## Abstract

Statin-associated muscle symptoms range from mild myalgia to severe myopathy and, rarely, immune-mediated necrotizing myopathy (IMNM). We report the case of a 62-year-old woman who had been receiving rosuvastatin 20 mg daily for approximately four months and presented to an outpatient clinic with four weeks of progressive fatigue, generalized myalgia, proximal muscle weakness, and difficulty walking. Initial evaluation revealed marked hyperCKemia, with a creatine kinase (CK) level of 8,586 U/L, elevated C-reactive protein (CRP) and lactate dehydrogenase (LDH) levels, transaminitis, and preserved renal function. Physical examination demonstrated diffuse muscle tenderness and reduced strength involving both the upper and lower extremities without focal neurologic deficits. Rosuvastatin was discontinued, and the patient was urgently referred to the emergency department for further evaluation and stabilization, with additional rheumatology and endocrinology referrals. According to the patient’s family, she was hospitalized for four days; however, inpatient records and treatment details were not available to the author. Following this family-reported hospitalization, she continued specialist care outside the author’s clinic. Serial laboratory results available through day 54 demonstrated persistent CK elevation, peaking at 9,239 U/L, followed by a gradual decline to 7,018 U/L and 3,550 U/L, while renal function remained preserved. The author could not determine whether confirmatory investigations were subsequently performed or verify the patient’s later treatment, recovery of muscle strength, final specialist diagnosis, or long-term clinical outcome from the available records. This case highlights the importance of early CK testing in statin-treated patients presenting with progressive weakness and emphasizes that aminotransferase elevation in this setting may reflect skeletal muscle injury rather than primary hepatic disease. Early recognition, prompt statin discontinuation, emergency evaluation when clinically indicated, and timely specialist referral are essential to reduce the risk of complications.

## Introduction

Statins are widely prescribed for dyslipidemia and cardiovascular risk reduction. Although they are generally well tolerated, statin-associated muscle symptoms remain among the most common reasons for treatment interruption and may range from self-limited myalgia to severe myopathy and rhabdomyolysis [1,2]. Creatine kinase (CK) is an intracellular enzyme that helps maintain rapid energy availability in skeletal muscle, and serum CK rises when muscle-fiber injury releases the enzyme into the circulation. Statin-associated muscle injury is multifactorial and may involve altered muscle-cell energetics, membrane instability, and, in a small subset of patients, immune-mediated injury [1,2].

Marked CK elevation with progressive weakness should prompt urgent evaluation for severe statin-associated myopathy and alternative inflammatory myopathies. A particularly important diagnostic consideration is statin-associated immune-mediated necrotizing myopathy (IMNM), which may present with proximal weakness, persistent CK elevation, and antibodies against 3-hydroxy-3-methylglutaryl-coenzyme A reductase (anti-HMGCR) [3-5]. Persistent marked CK elevation after statin discontinuation should prompt evaluation for anti-HMGCR antibody-associated IMNM. However, this diagnosis should not be considered confirmed without supportive serology and, when clinically indicated, electromyography, imaging, or muscle biopsy [3-5].

We present a case of severe rosuvastatin-associated myopathy recognized in the outpatient setting, complicated by marked hyperCKemia, transaminitis, and persistent CK elevation. This case highlights an important outpatient diagnostic challenge: distinguishing severe toxic statin myopathy from possible immune-mediated disease when CK remains markedly elevated after statin withdrawal and confirmatory investigations and long-term outcome data are not available to the initial clinician.

## Case presentation

A 62-year-old woman presented to an outpatient clinic with approximately four weeks of progressive fatigue, generalized muscle pain, weakness, and increasing difficulty rising from bed and walking. Her medical history included hypothyroidism, cerebrovascular accident with residual visual sequelae, prior deep venous thrombosis of the left lower extremity, type 2 diabetes mellitus, dyslipidemia, and prior right lower-extremity varicose vein surgery. Her medications included levothyroxine 125 mcg daily, metformin 750 mg, and rosuvastatin 20 mg daily, which she had been taking for approximately four months before presentation.

At the first outpatient visit, vital signs were stable, including blood pressure of 129/77 mmHg and a pulse of 87 beats per minute. Cardiopulmonary examination was normal, and neurologic examination did not reveal focal deficits. Given the patient’s progressive weakness, generalized myalgia, and history of hypothyroidism and statin exposure, laboratory evaluation was requested, including complete blood count, renal function, hepatic profile, inflammatory markers, thyroid function, urinalysis, and CK.

At a second outpatient visit, the patient returned with the requested laboratory results and ultrasound findings.

Initial laboratory testing demonstrated severe hyperCKemia with CK of 8,586 U/L. C-reactive protein (CRP) was elevated at 150.6 mg/L, erythrocyte sedimentation rate (ESR) was 66 mm/hour, lactate dehydrogenase (LDH) was 437 U/L, aspartate aminotransferase (AST) was 219 U/L, and alanine aminotransferase (ALT) was 210 U/L. Serum creatinine was preserved at 0.48 mg/dL. Hemoglobin was 11.7 g/dL, hematocrit was 34.9%, and platelet count was 437,000/mm^3^. Thyroid testing showed a thyroid-stimulating hormone (TSH) of 3.5 µIU/mL and a free thyroxine (T4) of 1.4 ng/dL, arguing against overt hypothyroidism as the sole explanation for the degree of CK elevation.

On examination at the second outpatient visit, the patient had diffuse muscle tenderness and decreased strength involving both the upper and lower extremities. Cardiopulmonary examination remained normal, and no focal neurologic deficits were identified.

**Figure 1:**
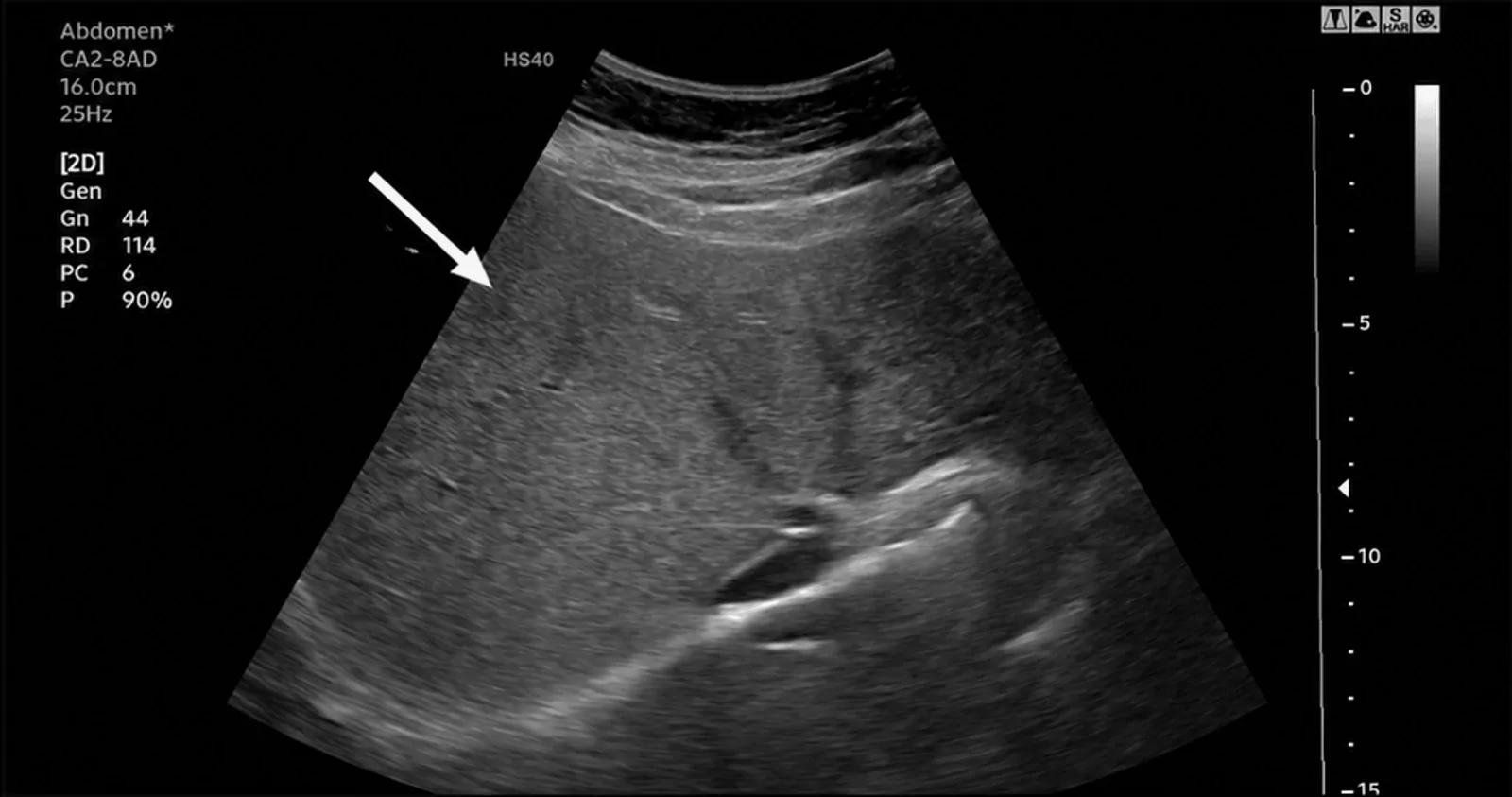
Abdominal ultrasound demonstrating hepatic steatosis Abdominal ultrasound obtained from the patient presented in this case. The arrow indicates the hepatic parenchyma demonstrating diffuse increased echogenicity consistent with hepatic steatosis

**Figure 2:**
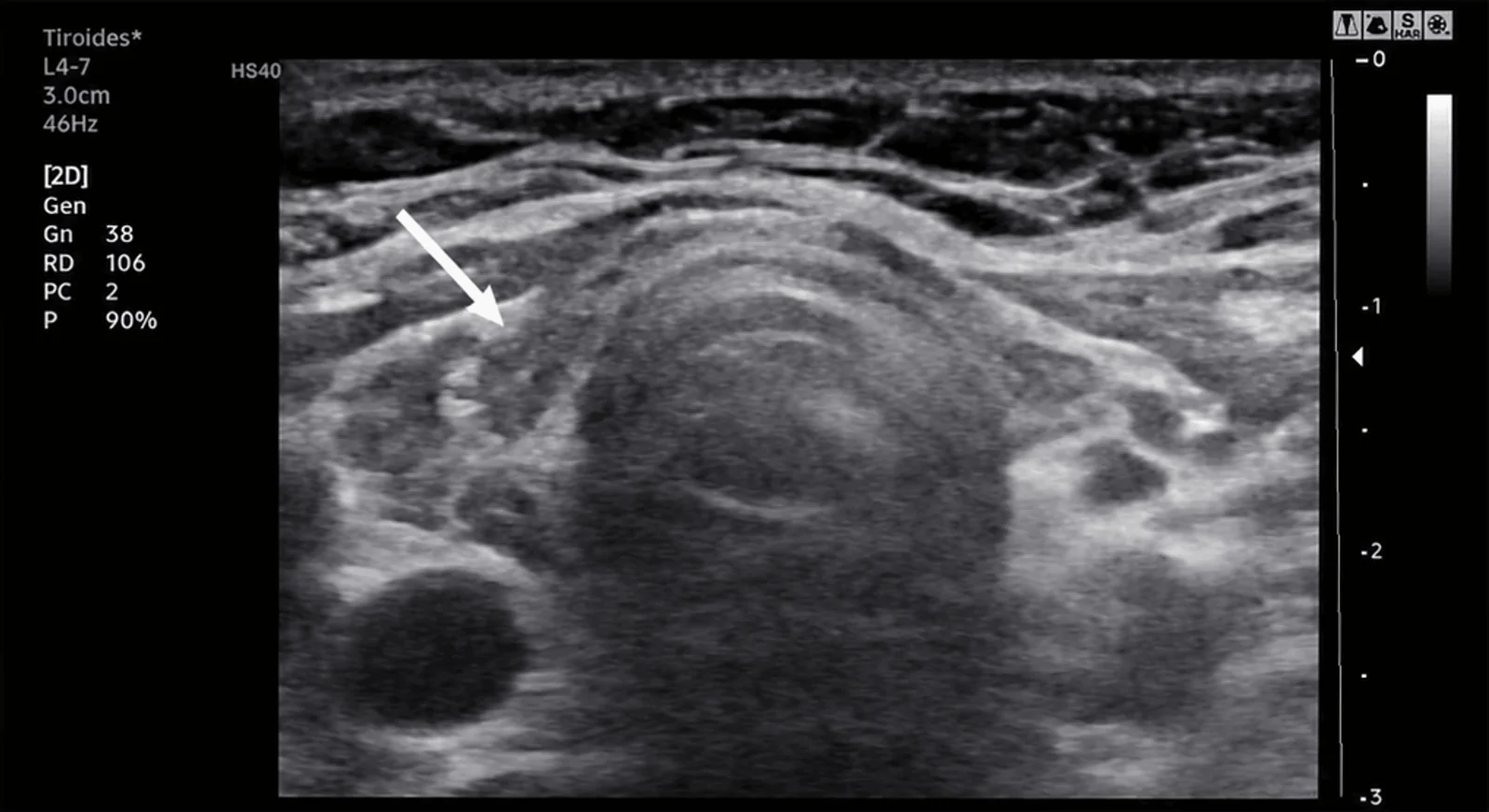
Thyroid ultrasound consistent with chronic thyroiditis Thyroid ultrasound obtained from the patient presented in this case. The arrow indicates the heterogeneous thyroid parenchyma consistent with chronic thyroiditis

Abdominal ultrasound demonstrated hepatic steatosis (Figure 1), and thyroid ultrasound was consistent with chronic thyroiditis (Figure 2).

Given the marked CK elevation, progressive weakness, statin exposure, and elevated inflammatory markers, severe rosuvastatin-associated myopathy was suspected. The differential diagnosis included toxic statin myopathy, statin-associated immune-mediated inflammatory myopathy, polymyositis, and hypothyroid-related myopathy. At the second outpatient visit, rosuvastatin was discontinued, urgent rheumatology and endocrinology referrals were arranged, and autoimmune testing was requested, including antinuclear antibody (ANA), antineutrophil cytoplasmic antibody (ANCA), anti-Jo-1 antibody, anti-Mi-2 antibody, and anti-SRP antibody. Because of the marked CK elevation, progressive weakness, and the risk of complications, the patient was also urgently referred to the emergency department of a public hospital for further evaluation and stabilization. According to information subsequently provided by the patient’s family, she was hospitalized for four days. The author did not have access to the hospital records and therefore could not verify the investigations performed, treatment administered, or clinical response during admission.

After discharge, the patient continued specialist care within the public hospital system and did not return to the author’s outpatient clinic. Subsequent laboratory tests through day 54 were ordered by other treating physicians at other clinical facilities but were performed by the laboratory associated with the author’s clinic; therefore, the results remained accessible in the clinic record. The author could not verify the subsequent specialist management, the completion of the requested autoimmune testing, the recovery of muscle strength, final specialist diagnosis, or long-term clinical outcome.

Serial laboratory follow-up demonstrated persistent hyperCKemia with gradual improvement (Table 1). CK increased to 9,239 U/L on day 22 and then decreased to 7,018 U/L on day 38 and 3,550 U/L on day 54. AST and ALT peaked at 342 U/L and later decreased to 233 U/L and then 120 U/L. Renal function remained preserved throughout follow-up, with a creatinine of 0.54 mg/dL on day 54.

**Table 1:** Serial laboratory findings during the patient’s clinical course Serial laboratory testing demonstrated persistent hyperCKemia, followed by gradual improvement after the discontinuation of rosuvastatin. Renal function remained preserved throughout follow-up. Rosuvastatin was discontinued shortly after the day-1 laboratory results were reviewed at the second outpatient visit. Laboratory results obtained after specialist referral were ordered by other treating physicians but remained accessible because the tests were performed by the laboratory associated with the author’s clinic *The marked elevation in CRP and ESR on day 54 occurred in the setting of a culture-confirmed *Escherichia coli* urinary tract infection and should be interpreted in that clinical context CK, creatine kinase; AST, aspartate aminotransferase; ALT, alanine aminotransferase; CRP, C-reactive protein; ESR, erythrocyte sedimentation rate; NA, not available

Clinical day	CK (U/L)	AST (U/L)	ALT (U/L)	CRP (mg/L)	ESR (mm/hour)	Creatinine (mg/dL)
Day 1	8,586	219	210	150.6	66	0.48
Day 22	9,239	342	342	NA	13	NA
Day 38	7,018	233	233	16.1	NA	NA
Day 54	3,550	120	120	304.2*	120	0.54

On day 54, inflammatory markers again increased, with a CRP of 304.2 mg/L and an ESR of 120 mm/hour. Concurrent urinalysis and urine culture demonstrated urinary tract infection due to *Escherichia coli* with >100,000 colony-forming units (CFU)/mL, limiting the interpretation of inflammatory markers as direct markers of myositis activity at that time.

## Discussion

This case illustrates several important diagnostic challenges in the outpatient evaluation of progressive muscle weakness in a patient receiving statin therapy. The patient’s initial symptoms were nonspecific and could easily have been attributed to hypothyroidism, chronic fatigue, viral illness, diabetic complications, or musculoskeletal disorders. However, the presence of progressive functional decline, difficulty rising from bed, impaired ambulation, diffuse myalgia, and proximal muscle weakness warranted the measurement of serum creatine kinase (CK), leading to the early recognition of severe muscle injury [1,2,6,7].

The degree of CK elevation was markedly abnormal and far exceeded the range expected for uncomplicated statin-associated myalgia. In the setting of ongoing rosuvastatin therapy, this raised concern for severe statin-associated myopathy. The CK level subsequently increased from 8,586 U/L to 9,239 U/L by day 22 and remained markedly elevated at 3,550 U/L on day 54 despite statin withdrawal. Persistent hyperCKemia after statin discontinuation raises concern for anti-HMGCR antibody-associated IMNM, although a progressive decline in CK may also occur during recovery from severe toxic statin myopathy [3-5]. In this patient, the persistent elevation increased concern for immune-mediated disease and justified specialist evaluation, but the downward CK trend alone could not establish that diagnosis. Results of anti-HMGCR antibody testing, electromyography, muscle magnetic resonance imaging, and muscle biopsy were not available in the records accessible to the author, and it was unknown whether these investigations were subsequently performed within the public hospital system. Accordingly, this case is most appropriately described as severe rosuvastatin-associated myopathy with concern for an underlying immune-mediated inflammatory myopathy rather than confirmed IMNM [3-5].

Another important teaching point is the interpretation of aminotransferase elevation. Although AST and ALT were substantially elevated, bilirubin, alkaline phosphatase, gamma-glutamyl transferase (GGT), coagulation studies, and renal function remained preserved. In the context of severe hyperCKemia and progressive muscle weakness, the transaminitis was most likely attributable, at least in part, to skeletal muscle injury rather than primary hepatic disease. Recognizing this biochemical pattern is important because misattributing elevated aminotransferases solely to liver pathology may delay the diagnosis of severe statin-associated muscle injury [1,2,6,7].

This case also demonstrates why inflammatory markers should be interpreted within the broader clinical context. Although the initial elevation in CRP and ESR supported an inflammatory process, the subsequent marked increase coincided with a culture-confirmed *Escherichia coli* urinary tract infection. Therefore, the later inflammatory marker elevation cannot be confidently attributed to ongoing myositis activity alone.

Limitations of this case include the availability of only two direct outpatient evaluations and the absence of access to hospitalization and subsequent specialist consultation records. Although laboratory results through day 54 remained accessible because tests ordered by other treating physicians were performed by the laboratory associated with the author’s clinic, the patient’s subsequent treatment, recovery of muscle strength, final specialist diagnosis, and long-term clinical outcome could not be verified. Autoimmune testing was recommended at the second outpatient visit, but it could not be confirmed whether the requested tests were completed. It was also unknown whether anti-HMGCR antibody testing, electromyography, muscle magnetic resonance imaging, or muscle biopsy was subsequently performed. These limitations precluded definitive etiologic classification. Long-term clinical and laboratory follow-up is important to document recovery and exclude persistent immune-mediated disease.

Despite these limitations, this case reinforces the importance of maintaining a high index of suspicion for severe statin-associated myopathy in patients presenting with progressive proximal muscle weakness while receiving statin therapy. Early CK measurement, the prompt discontinuation of the offending medication, and appropriate specialist referral are essential to reduce morbidity and facilitate the timely diagnosis of potentially life-threatening muscle disease [1-7].

## Conclusions

This single case highlights that severe statin-associated myopathy should be considered in statin-treated patients presenting with progressive myalgia, proximal weakness, and functional decline. Early CK testing and the prompt discontinuation of the statin are essential. Persistent hyperCKemia after statin withdrawal warrants specialist assessment and confirmatory evaluation for immune-mediated disease, including anti-HMGCR antibody testing, together with continued clinical and laboratory follow-up to document CK normalization and the recovery of muscle strength. Aminotransferase elevation in this setting may reflect skeletal muscle injury and should be interpreted alongside CK, bilirubin, GGT, alkaline phosphatase, and renal function.

## References

[1] Thompson PD, Clarkson P, Karas RH (2003). Statin-associated myopathy. JAMA.

[2] Stroes ES, Thompson PD, Corsini A (2015). Statin-associated muscle symptoms: impact on statin therapy-European Atherosclerosis Society Consensus Panel Statement on assessment, aetiology and management. Eur Heart J.

[3] Mammen AL (2016). Statin-associated autoimmune myopathy. N Engl J Med.

[4] Allenbach Y, Benveniste O, Stenzel W, Boyer O (2020). Immune-mediated necrotizing myopathy: clinical features and pathogenesis. Nat Rev Rheumatol.

[5] Khoo T, Chinoy H (2023). Anti-HMGCR immune-mediated necrotising myopathy: addressing the remaining issues. Autoimmun Rev.

[6] Selva-O'Callaghan A, Alvarado-Cardenas M, Pinal-Fernández I (2018). Statin-induced myalgia and myositis: an update on pathogenesis and clinical recommendations. Expert Rev Clin Immunol.

[7] Rosenson RS, Baker SK, Jacobson TA, Kopecky SL, Parker BA (2014). An assessment by the Statin Muscle Safety Task Force: 2014 update. J Clin Lipidol.

